# Prognostic and Clinical Significance of miRNA-205 in Endometrioid Endometrial Cancer

**DOI:** 10.1371/journal.pone.0164687

**Published:** 2016-10-13

**Authors:** Milosz Wilczynski, Justyna Danielska, Monika Dzieniecka, Bozena Szymanska, Michal Wojciechowski, Andrzej Malinowski

**Affiliations:** 1 Department of Operative Gynecology, Endoscopy and Gynecologic Oncology, Polish Mother’s Memorial Hospital Research Institute, Lodz, Poland; 2 Radiotherapy Department, Medical University in Lodz, Lodz, Poland; 3 Department of Pathology, Polish Mother’s Memorial Hospital Research Institute, Lodz, Poland; 4 The Central Laboratory of Medical University in Lodz, Lodz, Poland; 5 Department of Surgical and Endoscopic Gynecology, Medical University in Lodz, Lodz, Poland; Seoul National University College of Pharmacy, REPUBLIC OF KOREA

## Abstract

Endometrial cancer is one of the most common malignancies of the reproductive female tract, with endometrioid endometrial cancer being the most frequent type. Despite the relatively favourable prognosis in cases of endometrial cancer, there is a necessity to evaluate clinical and prognostic utility of new molecular markers. MiRNAs are small, non-coding RNA molecules that take part in RNA silencing and post-transcriptional regulation of gene expression. Altered expression of miRNAs may be associated with cancer initiation, progression and metastatic capabilities. MiRNA-205 seems to be one of the key regulators of gene expression in endometrial cancer. In this study, we investigated clinical and prognostic role of miRNA-205 in endometrioid endometrial cancer. After total RNA extraction from 100 archival formalin-fixed paraffin-embedded tissues, real-time quantitative RT-PCR was used to define miRNA-205 expression levels. The aim of the study was to evaluate miRNA-205 expression levels in regard to patients’ clinical and histopathological features, such as: survival rate, recurrence rate, staging, myometrial invasion, grading and lymph nodes involvement. Higher levels of miRNA-205 expression were observed in tumours with less than half of myometrial invasion and non-advanced cancers. Kaplan-Maier analysis revealed that higher levels of miRNA-205 were associated with better overall survival (p = 0,034). These results indicate potential clinical utility of miRNA-205 as a prognostic marker.

## Introduction

Endometrial cancer (EC) is one of the most common malignancies among women worldwide. It is estimated that EC is the second most frequent malignant tumour of the female reproductive tract, just after cervical cancer. In 2012, approximately 320 000 newly diagnosed cases were reported globally. Due to the longer expected life duration, lifestyle diseases and increase of obesity rates, EC has become a major oncological issue in developed countries [1].

Bokhman’s dualistic model divides EC into two distinct pathogenetic categories. Clinical, pathological and molecular evidence shows that EC may be categorized into two types: type I (estrogen-dependent) and type II (estrogen-nondependent). Type I is the more common subtype, occurring in about 70–80% of EC cases. In terms of histopathology, it corresponds to endometrioid endometrial cancer (EEC) and usually consists of low grade and low stage tumours with favourable prognosis [2]. In contrast, type II tumours occur less frequently and are represented by clear-cell and serous carcinomas. This subtype of EC is composed of poorly differentiated tumours that are characterised by unfavourable prognosis [2,3]. Type I EC is characterised by *PTEN*, *KRAS*, *CTNNB1*, *PIK3CA* mutations and microsatellite instability (MSI). Molecular data suggests that EEC are highly mutated in regard to PI3K/AKT/mTOR and Wnt/beta-catenin signaling pathways [4]. Molecular studies proved that type II EC shows distinct mutational abnormalities, such as *HER2* amplification and *TP53* mutations [4].

However, it seems that some endometrioid tumours share similar clinical features as type II cancers, developing more aggressive disease, forming distant metastases and being characterised by less favourable prognosis [5,6]. Despite the fact that histopathological evaluation and staging of EC is crucial, new molecular prognostic biomarkers are required to better predict the possible outcome. Such specific and sensitive markers might help to differentiate between high and low risk EEC.

MiRNAs are small, non-coding RNA molecules of 18–25 nucleotides [7]. MiRNAs are transcribed on basis of genes that are scattered in the whole human genome. MiRNAs take part in epigenetic and, most importantly, post-transcriptional regulation of gene expression. RISC (RNA-induced silencing complex) is a multiprotein complex that is responsible for RNA cleavage. The RISC complex activation is triggered by miRNAs incorporation. Through binding to the 3′-untranslated region (3′-UTR) of target mRNAs, miRNAs can cause mRNA cleavage, mRNA decay or translational repression [8,9]. Thanks to that, miRNAs may play an important role in many critical cellular processes including apoptosis, proliferation and cell differentiation [10,11]. Molecular characteristics of miRNA allow for not entirely complementary binding to the target mRNAs. One miRNA might possibly produce an effect on expression of many genes [12]. Review of recent literature suggests that miRNAs may play an important role in carcinogenesis and cancer progression. Aberrant miRNA expression has been reported in a variety of human malignancies, including EC tissues [13]. MiRNAs seem to be ideal for molecular biology studies, due to its robust, stable features and presence in FFPE tissues [14]. MiRNAs’ attributes indicate its’ potential role as a prognostic biomarker. However, existing data concerning prognostic role of miRNAs in EC is scarce. Recently, several reports evaluating the expression profiles of miRNAs in endometrial cancer have been published, however, there is still insufficient information about its’ potential role as prognostic factors [13, 15, 16, 17]. Elevated levels of miRNA-205 in EC tissues were demonstrated by some authors [18]. There is evidence that miRNA-205 may promote invasion and tumour proliferation due to its potential influence on ESRRG (estrogen-related receptor γ) [19]. On the other hand, other studies emphasize that miRNA-205 may interfere with PTEN, a well-known tumour suppressor that is inactivated in many types of malignancies, including EC [20,21]. Furthermore, miRNA-205 in association with transcriptional factors for E-cadherin–ZEB1 and ZEB2 (Zinc finger E-box-binding homeobox 1/2)–may regulate EMT process (epithelial-mesenchymal transition) [22]. EMT, a process in which epithelial cells gain invasive abilities, might be critical to the initiation of metastases.

MiRNA-205 seems to be one of the key-regulators of EC carcinogenesis and tumour promotion, however, molecular basis of its importance still has to be elucidated. Therefore, we decided to investigate clinical and prognostic significance of miRNA-205 in endometrioid endometrial cancer.

## Methodology and Patients

Ethical approval for the study was obtained from Polish Mother’s Memorial Hospital Research Institute Ethics Committee and all participants provided their written consent.

**Fig 1:**
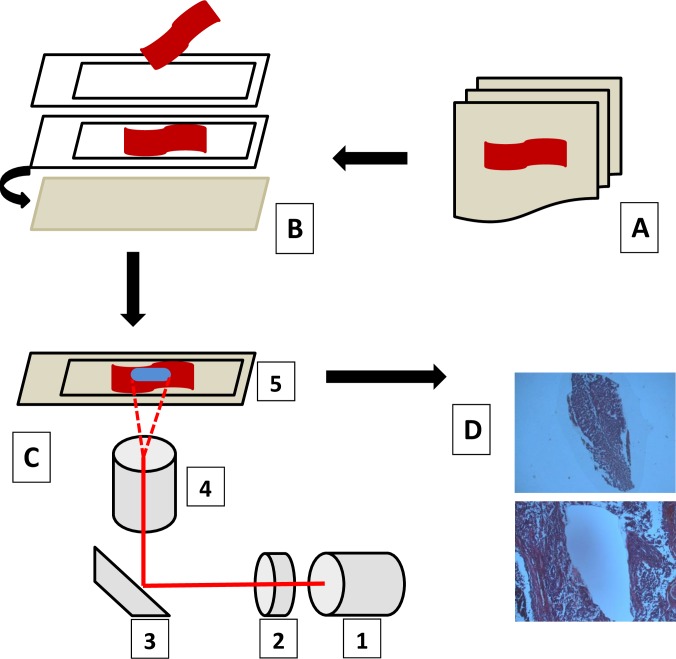
Laser microdissection technique–general overview showing basic steps, according to MMI CellCut system protocol (A–selection of paraffin embedded tissue blocks and sectioning of blocks; B–placing sections on membrane-slides which are protected by glass slides; C–selection of tissue and cutting with laser: 1 –UV laser, 2 –focusing lenses, 3- polarization beam combiner, 4 –objective, 5 –membrane-slide with tissue section; D–final product, microdissected tissues may be collected by an adhesive cap).

Ninety EEC patients, who underwent laparoscopic or abdominal surgery between 2002 and 2014, were selected. Total hysterectomy with bilateral salpingo-oophorectomy and pelvic lymphadenectomy was performed in all cases, which assured precise tumour staging. Adjuvant treatment was introduced in most of the cases. It was omitted when IA G1 tumour (without any additional risk factors) was diagnosed or general patients’ condition was unsatisfactory. Clinical information was acquired from medical records. Additionally, a follow-up of all patients was carried out in the out-patient clinic of Polish Mother’s Memorial Hospital Research Institute. Information concerning age, adjuvant treatment applied, tumour recurrence and survival were obtained. The most important clinical and pathological characteristics of patients are provided in Tables 1 and 2. MiRNA-205 expression levels were evaluated in archival FFPE (formalin-fixed, paraffin-embedded) EC tissues. A precise identification of endometrial cancer areas was performed by an experienced pathologist. Only EEC tissues were selected, ambiguous and mixed tumours were excluded from the study. Careful micro dissection of EEC samples was performed in order to minimalize the risk of contamination with noncancerous tissues (Cellcut Plus MMI, Fig 1). The amount of samples in the reference group was dependent on the reproducibility and consistency of miRNA-205 expression levels. The reference group consisted of ten normal endometrium samples that were retrieved after hysterectomy for benign diseases.

**Table 1:** Clinical characteristics.

Characteristics	n/quantity	%
Mean age (range)	64.3 (37–84)	
BMI>25	70	77.8
Selected comorbidities:		
Ischaemic heart disease	22	24.4
Hypertension only	38	42.2
Diabetes only	3	3.3
Diabetes and hypertension	18	20
Treatment		
Adjuvant treatment	71	78.9
No adjuvant treatment	19	21.1
Brachytherapy only	14	15.5
Teleradiotherapy only	1	1.1
Brachytherapy and teleradiotherapy	49	54.4
Chemotherapy only	3	3.3
Chemotherapy and radiotherapy	3	3.3
Hormonotherapy only	1	1.1
Recurrence	18	0.2
Deaths	20	22.2
Related to comorbidities	2	2.2
Related to EC	18	0.2
Follow-up period–mean time (range)	48.85 (2–119)	
Follow-up period after excluding cases of death–mean time (range)	59 (16–119)	
Overall survival in months–mean time (range)	46.6 (2–119)	

**Table 2:** Pathological characteristics.

Characteristics	n/quantity	%
FIGO stage		
IA	25	27,8
IB	24	26,7
II	13	14,4
IIIA	4	4.4
IIIB	5	5.6
IIIC	12	13.3
IVA	0	0
IVB	7	7.8
Grading		
G1	34	37.8
G2	42	46.7
G3	14	15.5
Myometrial infiltration <0.5	36	40
Myometrial infiltration ≥ 0.5	54	60
Positive lymph nodes	15	16.7

RNA was isolated with the use of miRNeasy FFPE Kit (Qiagen), according to the manufacturer’s protocol. In brief, all samples were deparaffinised (Deparaffinization Solution–Qiagen), digested with proteinase K (followed by heat treatment). Supernatant was treated with DNase, then mixed with Buffer RBC. 100% ethanol was used to adjust binding conditions. Subsequently, the sample was transferred to RNeasy MinElute Spin Column and elution with RNase-free water was performed. Total mRNA concentrations were evaluated with spectrophotometry (PicoDrop) and the quality of samples was validated based on the ratio of absorptions at 260 nm vs 280 nm. The next step of the procedure was cDNA synthesis. Reverse transcription was carried out by means of TaqMan® MicroRNA Reverse Transcription Kit (Applied Biosystems) and microRNA-specific primers (RT primer), according to manufacturer’s protocol. Specific reagents for miRNA-205 and endogenous control (RNU6B) were employed in real time polymerase chain reaction (TaqMan® MicroRNA Assays—Applied Biosystems). Applied Biosystems 7900HT Fast Real-Time PCR System machine was used for real time PCR evaluation, according to manufacturer’s recommendations. Samples were run in triplicate. All data was analyzed by Sequence Detection System 2.4 (Applied Biosystems). Relative expression was calculated according to the Ct method 2^-ΔΔCt^.

### Statistical analysis

Kaplan-Meier survival curves were used to analyse the relationship between the expression levels of miRNA-205 and patients’ survival rate. The differences between studied groups were determined by Mann–Whitney U or Kruskal–Wallis test. Spearman's rank correlation coefficient was used in order to measure the statistical dependence between two variables. Statistical significance was set at p<0.05.

## Results

**Fig 2:**
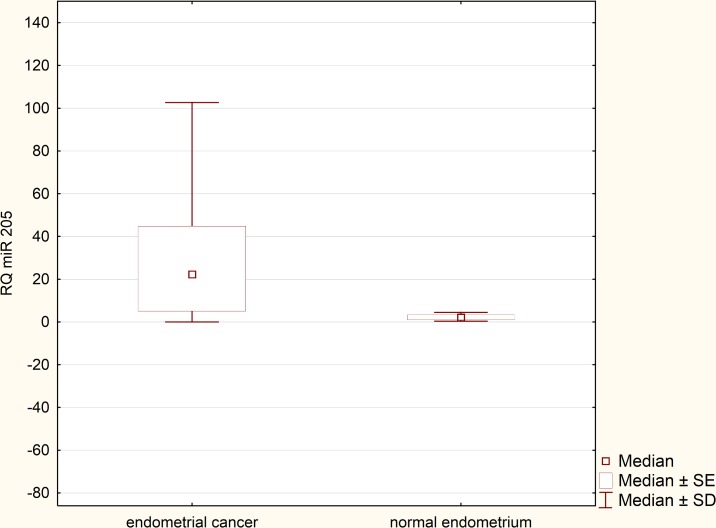
MiRNA-205 expression in normal and endometrioid endometrial cancer tissue samples (Mann Whitney U test, p = 0,000158).

**Fig 3:**
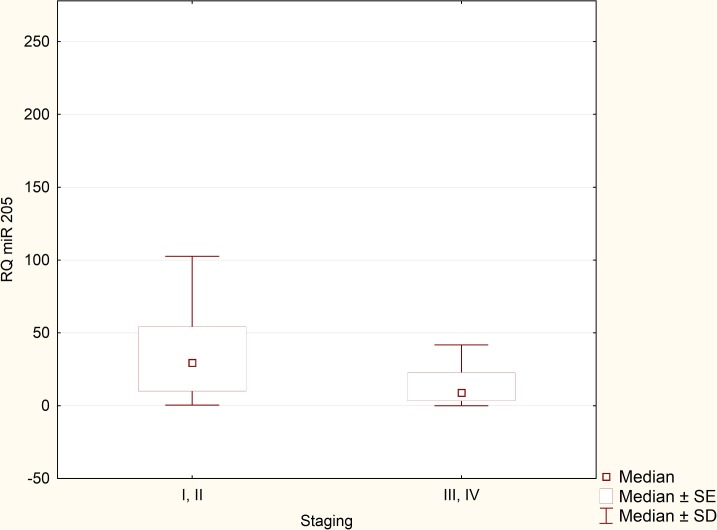
MiRNA-205 expression: I, II vs. III, IV FIGO stage (p = 0,045).

**Fig 4:**
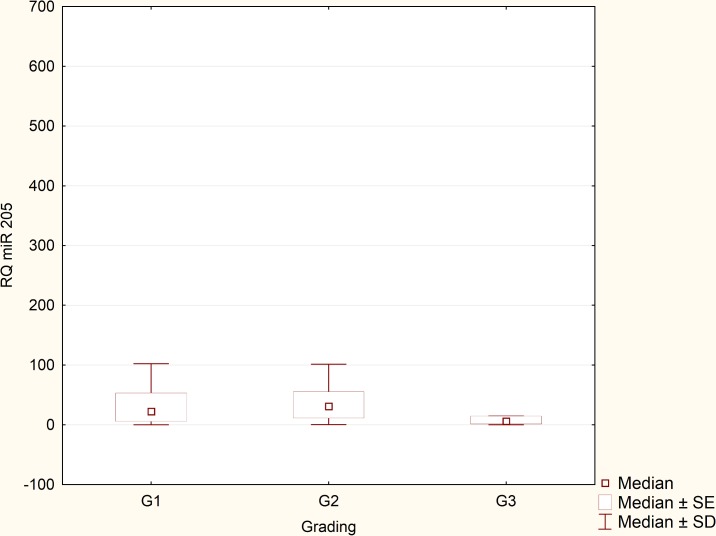
MiRNA-205 expression in terms of grading: G1 vs. G2 vs. G3 (Kruskal–Wallis test, G2 vs. G3, p = 0.02).

**Fig 5:**
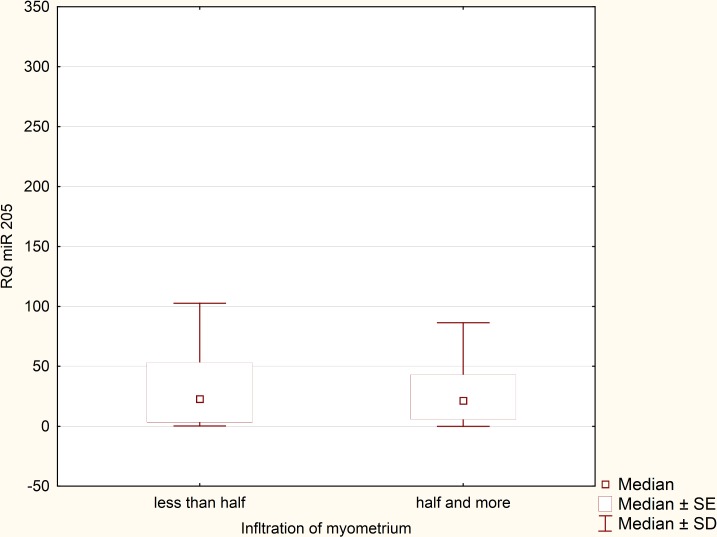
MiRNA-205 expression: < ½ myometrial invasion vs. ≥ ½ myometrial invasion (Mann-Whitney U test, p = 0,03).

**Fig 6:**
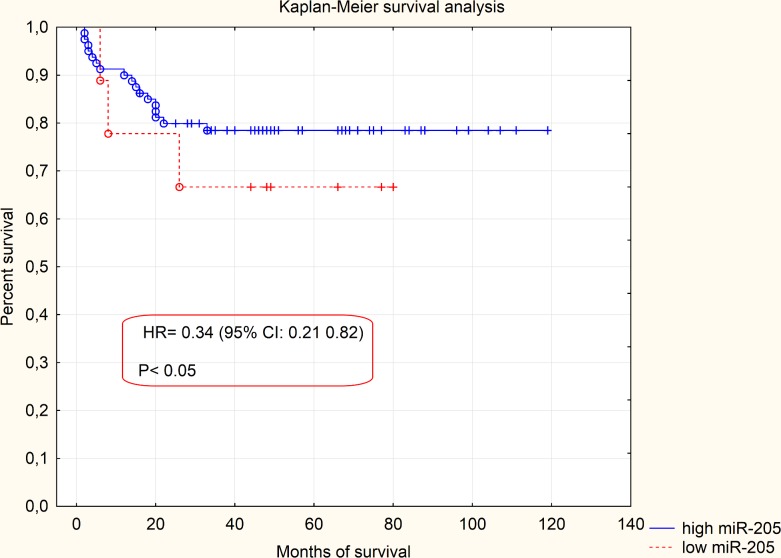
Kaplan-Meier survival analysis based on miRNA-205 expression values (cut-off expression value ΔΔCt = 0.4). High expression of miRNA-205 vs. low expression of miRNA-205 (HR 0,34, 95% CI 0.21–0.82; log-rank test, p = 0,034).

Expression levels of miRNA-205 in the reference group (normal endometrium) were extremely reproducible and remained relatively low in all samples. After performing statistical analysis, it turned out that there was no need to exceed the number of samples in the reference group. Expression of miRNA-205 was greatly upregulated in the EEC specimens in comparison to normal endometrium samples (p = 0,000158, Fig 2). RQ values, which were evaluated in the EEC tissues derived from 90 patients, were used to determine miRNA-205 clinical significance. Relationship between miRNA-205 expression and various clinicopathological factors was verified. We found no statistically significant correlation between miRNA-205 expression and presence of metastatic lymph nodes (p = 0,41) and recurrence rate (p = 0.51). Likewise, no significant correlation was detected in terms of FIGO stages. Therefore, we divided the patients into two clinical subgroups. The first clinical subgroup consisted of early stage EEC patients, namely I and II FIGO stage. In opposite, the second clinical subgroup was formed by late stage EEC patients (III and IV FIGO stage). Statistical analysis showed that miRNA-205 expression maintained at higher levels in early stage EEC patients (p = 0.045, Fig 3). Furthermore, we identified correlation between miRNA-205 expression and grading (according to the scheme: G1 –low grade tumours, G2 –intermediate grade tumours, G3 –high grade tumours). We found that miRNA-205 expression tended to be lower in poorly differentiated (G3) tumours in comparison to moderately differentiated tumours (G2) (p = 0.02, Fig 4). To further determine clinical significance of miRNA-205 in EEC, we separated the patients into two additional clinical subgroups based only on the depth of myometrial infiltration (irrespective of metastatic or cervical status). Patients affected by less than half myometrial invasion were assigned to the first subgroup and patients with invasion equal to or more than half of the myometrium formed the second subgroup. Our analysis demonstrated that higher miRNA-205 expression levels were characteristic of the first clinical subgroup (p = 0.039, Fig 5). To determine prognostic significance of miRNA-205 we used Kaplan-Meier estimator. We introduced two clinical subgroups that consisted of patients with high and low levels of miRNA-205 (miRNA-205 expression cut-off values - ΔΔCt = 0.4). The analysis indicated that miRNA-205 expression levels were considerably associated with patient survival. Higher levels of miRNA-205 were characteristic of better survival among patients. Patients with lower levels of miRNA-205 tended to have worse survival (HR 0.34–95% CI 0.21–0.82, log-rank test, p = 0.034, Fig 6).

## Discussion

Since miRNA was discovered, numerous studies have proven that there is a difference between normal and cancerous tissues in terms of miRNA expression levels. Several authors demonstrated that it is also true for endometrial cancer, however, just a few miRNAs were reported to be consistently upregulated throughout the studies. Based on studies that have been published so far, miRNA-185, miRNA-106a, miRNA-181a, miRNA-210, miRNA-423, miRNA-103, miRNA-107, miRNA-let7c, miRNA-205, miRNA-449 and miRNA-429 were found to be upregulated in endometrial cancer compared to normal tissues [23]. We decided to choose miRNA-205 for our study as it might be one of key regulators in endometrial cancer carcinogenesis and progression. Molecular evidence indicate that miRNA-205 presents conflicting functions in different types of cancers. MiRNA-205 may counteract the process of epithelial-mesenchymal transition (EMT) by targeting PKCε (protein kinase C epsilon type) or ZEB2 (zinc finger E-box-binding homeobox 2) [22]. In breast cancer, miRNA-205 targets VEGF-A (vascular endothelial growth factor A) which plays an important role in cancer metastasis [24]. On the other hand, miRNA-205 exerts oncogenic effect targeting PTEN (phosphatase and tensin homolog) or ESRRG estrogen-related receptor gamma (ESRRG) [25,26].

One of the objectives of this study was to demonstrate that EEC has a different expression of miRNA-205 compared to healthy endometrium. This concept was a basis for evaluation of miRNA-205 expression in the context of clinicopathological data and patients survival. Due to the fact that type I and II EC are characterised by different genetic aberrations, we decided to focus only on EEC. Several previous studies by other authors were based on heterogeneous groups of patients with different histological diagnosis, including serous, clear-cell carcinomas, malignant mixed mullerian tumours/carcinosarcomas [27, 28, 29]. In our study we proved that miRNA-205 is over-expressed in EEC tissue specimens in comparison to normal endometrium. This conclusion seems to be consistent with findings of other authors. Cohn et al. investigated miRNA expression profiles in 141 EC patients. Authors reported that miRNA-205 was significantly upregulated in EC tissues (besides 4 other miRNAs: -200c, -183, -223, -425) [27]. Torres et al. performed qRT-PCR analysis of 104 samples proving that miRNA-205 was upregulated in EEC tissue. Signature of 3 miRNAs (miR-92a/miR-205/miR-410) classified tumour tissues with high accuracy (AUC = 0.984) [29]. Use of Next Generation Sequencing (NGS) also allowed for identification of higher miRNA-205 expression levels in type I EC tissue and plasma samples. Tsukamoto et al. additionally evaluated preoperative and postoperative miRNA plasma expression profiles in patients who underwent hysterectomy [30]. MiRNA-205 concentration significantly decreased after the surgery. It is worth mentioning that miRNA-205 dysregulation has been observed in other types of cancers, as well. MiRNA-205 was reported to be upregulated in breast carcinomas, whereas, downregulated in prostate cancers [31,32]. MiRNA-205 dysregulation has not been reported by all authors. Cohn et al. demonstrated presence of 18 dysregulated miRNAs in EC tissues derived from 141 patients. The study was conducted in 121 early stage EEC patients and 20 patients with advanced carcinoma of heterogeneous histology. Despite the inclusion of many cases, authors did not report miRNA-205 aberrant expression [27].

Clinical role of miRNA-205 in endometrial cancer has not been elucidated so far. Few authors attempted to evaluate miRNA-205 expression levels in regard to such characteristics as myometrial infiltration, grading, relapse, FIGO stage or nodal metastasis. In majority, those studies were based on small groups of patients, which resulted in interpretation difficulties and less reliable conclusions. Su et al. included in their study 53 cases of EEC showing upregulation of miRNA-205 in cancerous tissues, however, no correlations were found between miRNA expression level and clinicopathological data [26]. Chung et al. identified a set of aberrant miRNA in 30 EEC samples from Honk Kong women [13]. Apart from other miRNAs, authors evaluated miRNA-205 dysregulation with reference to clinicopathological data. Upregulation of miRNA-205 was associated with advanced stage, myometrial invasion and recurrence. Above mentioned results are discordant with our findings. We encountered higher miRNA-205 expression levels in cases of less than half myometrial invasion and early stages of EEC (I and II FIGO stage). No correlation was found between miRNA-205 upregulation and recurrence rate. Lack of similarity in the results may be related to the fact that Chung et al. based their study on barely 30 patients. Furthermore, racial disparity may also influence the outcome. It is hypothesised that dysregulation of miRNAs may be associated with racial molecular diversity. For instance, Maxwell et al. found miRNA racial differences in white and black patients [33]. Tsukamoto et al., with the use of NGS, identified a group of miRNA characteristic of EEC and evaluated their clinical significance [30]. Authors found that miRNA-205 was upregulated in cases of poorly differentiated tumours. Our results showed a different tendency indicating that miRNA-205 expression levels were lower in poorly differentiated (G3) tumours in comparison with moderately differentiated tumours (G2). However, conclusion made by Tsukamoto et al. was based on merely two cases of grade 3 endometrial cancers.

Prognostic significance of miRNA-205 in malignant neoplasms has been an issue for several years now. The predictive value of miRNA-205 was evaluated mainly in prostate, breast and lung cancers. Results of meta-analysis, which was based on 17 studies, showed that elevated miRNA-205 expression levels were associated with enhanced overall survival in breast cancer and superior disease-free survival/recurrence-free survival in the adenocarcinoma subgroup [34]. However, this meta-analysis included only one study devoted to the prognostic role of miRNA-205 in endometrial cancer, published by Karaayvaz et al. [25]. According to the results of the study by Karaayvaz et al., miRNA-205 expression levels were significantly associated with overall survival. They reported that patients with higher miRNA-205 levels tended to have worse prognosis. In our study, we also found significant upregulation of miRNA-205 and divided EEC samples into two subcategories: samples with lower and higher miRNA-205 overexpression levels. Results of the present study showed that patients with lower overexpression levels of miRNA-205 were characterised by less favourable prognosis. Therefore, there is a clear discordance between our study and findings by Karaayvaz et al. It is worth emphasizing that Karaayvaz et al. included 48 patients who were diagnosed with EC of heterogeneous histology (EEC, serous carcinoma, carcinosarcoma, clear cell carcinoma and undifferentiated carcinoma). Authors reported significant overexpression of miRNA-205 in endometrial cancer tissues in comparison with normal endometrium and suggested that it is not specific to histologic type. Such assumption seems to be oversimplification of the issue, as molecular/genetic diversity of endometrial cancer histologic subtypes is irrefutable [35]. Even if EC is generally characterised by miRNA-205 upregulation, it can be hypothesised that EC may be represented by different miRNA-205 overexpression levels that are specific to histology type.

Kaplan-Meier analysis showed that higher/lower miRNA-205 expression levels were associated with overall survival. However, we were surprised to find no correlation between miRNA-205 expression levels and such prognostic factors as the recurrence rate or presence of nodal metastases. Despite the fact that no correlation was found in terms of different FIGO stages, patients subdivision into early and late stages of cancer proved that higher miRNA-205 expression levels may be indicative of less advanced EC. Such discrepancy might be caused by relatively small number of patients with recurrent and metastatic disease in the study. Our results might also be dependent on the normalization strategy for real-time PCR analysis. Normalization is most commonly performed by using stably expressed endogenous controls which provide reproducible and reliable results. In our study, we decided to choose RNU 6B, small-nucleolar RNA, that is highly abundant in human tissues. RNU 6B is frequently recommended as a human endogenous control displaying minimal variability across different tissues [36]. However, recent studies have shown that RNU 6B may be substituted by even more stable endogenous controls. Torres et al. identified a set of endogenous controls that is suitable in endometrioid endometrial cancer tissues [37]. RNU48, U75, RNU44 were found to be stably expressed both in malignant and normal tissues.

MiRNA-205 may be one of the key factors determining tumor initiation, progression and cancer invasion. However, its exact molecular way of action has not been elucidated yet. Its role in malignancy remains controversial as miRNA-205 may act as a tumour suppressor or an oncogene, depending on the cancer type [38]. The epithelial-mesenchymal transition (EMT) is a vital process in which epithelial cells lose their polarity and adhesive capabilities. As a result, they gain migratory and invasive properties. EMT may play an important role in the promotion of cancer invasion and metastasis [39]. MiRNA-205 (together with miRNA-200 family) regulate expression of the E-cadherin transcriptional repressors ZEB1 and ZEB2, factors that play an important role in EMT. Gregory et al. discovered that miRNA-205 expression was downregulated in cells that had undergone EMT in response to transforming growth factor (TGF– β). This evidence shows that miRNA-205 inhibition may lead to EMT induction through upregulation of ZEB1/ZEB2 [22]. MiRNA-205 may be strictly associated with EMT process, which is an important step in EC progression. However, miRNA-205 is upregulated in both early stage and advanced EC [25,27,40]. Based on suppressive impact of miRNA-205 on EMT, one may expect that its high levels may favour better prognosis. In view of this argumentation, it can be hypothesised that higher levels of miRNA-205 may be a marker of an early stage disease and its overexpression relatively lowers along with tumour progression and possible EMT induction. This hypothesis may partially explain results of the present study, showing that higher levels of miRNA-205 are associated with more favourable prognosis.

MiRNA-205 may target other proteins that are involved in carcinogenesis and progression of EC. One of the direct miRNA-205 targets is *PTEN* suppressor gene, which inactivation is one of the mostly reported genetic aberrations in EEC (occurring in 37–61% of cases) [41]. It has already been proved that miRNA-205 negatively regulates PTEN expression, posttranscriptionally [25, 42]. Karaayvaz et al. showed that the expression of miRNA-205 and PTEN are inversely correlated in endometrial cancer. It is believed that PTEN inactivation occurs at an early stage of carcinogenesis as its lower expression was detected in endometrial hyperplasia [42]. In this view, higher miRNA-205 expression levels, that we found in cases of early stage disease, may be associated with negative PTEN regulation during initial steps of EC carcinogenesis.

In summary, our results indicate that higher miRNA-205 expression levels may be associated with better prognosis and less advanced stages of EC. Molecular basis of this phenomenon should be elucidated in further studies. The strengths of this study include homogenous population of only EEC patients and representative size of the research group that consisted of all FIGO stages. There are, however, limitations to our research. Clinical utility of miRNA-205 as a prognostic biomarker still requires additional validation based on larger cohorts of EC patients.
